# Crystal Structure and Thermoelectric Properties of Lightly Substituted Higher Manganese Silicides

**DOI:** 10.3390/ma11060926

**Published:** 2018-05-30

**Authors:** Yuzuru Miyazaki, Haruki Hamada, Hiroki Nagai, Kei Hayashi

**Affiliations:** Department of Applied Physics, Graduate School of Engineering, Tohoku University, 6-6-05 Aoba, Aramaki, Aoba-ku, Sendai 980-8579, Japan; ipheion.sail@gmail.com (H.H.); nagai@crystal.apph.tohoku.ac.jp (H.N.); hayashik@crystal.apph.tohoku.ac.jp (K.H.)

**Keywords:** higher manganese silicide, solid solution, incommensurate composite crystal, MnSi striation, thermoelectric properties, crystal structure, domain separation

## Abstract

The dissipation of MnSi layered precipitates during solidification is critical for further enhancement of the thermoelectric properties of the higher manganese silicides. We have investigated the effects of partial substitution of V in Mn sites and of Ge in Si sites on the crystal structures and thermoelectric properties of these silicides in detail. As previously reported, a small amount of V-substitution is quite effective in completely dissipating the MnSi striations; in contrast, a small proportion of these MnSi striations always remains present in the Ge-substitution case, even in the vicinity of the Ge solubility limits. For completely MnSi-dissipated samples, domain separation of the regular and highly strained arrangements of the Si atoms is realized. This domain separation suppresses the deterioration of the carrier mobility of the partially V-substituted samples and maintains even higher electrical conductivity to yield a high thermoelectric power factor of ∼2.3 mW/K${}^{2}$m at higher temperatures.

## 1. Introduction

Higher manganese silicides (HMSs) have been known to be potential thermoelectric (TE) materials since the 1960s [1,2,3]. While the dimensionless figure of merit, zT, which is the product of the figure of merit *z* multiplied by temperature *T*, is less than unity for these materials, HMSs have recently attracted renewed interest because they consist of naturally abundant elements that are less toxic than other TE materials and are thermally stable in the atmosphere. Several approaches have been used to enhance the TE performances of HMSs, including partial substitution of the Mn and/or Si sites [4,5,6,7,8,9], use of unconventional preparation methods [10,11] and modification of their micro/nanostructures [12,13,14]. As a result of these efforts, samples with zT> 1 have been successfully prepared using supersaturated 16% Re-substituted solid solutions for the Mn sites [11]; such samples would be impossible to obtain under equilibrium conditions.

In addition to the improvement of the zT values, another critical problem exists for use of these materials in practical applications: the formation of MnSi striations with sub-micron-order thicknesses upon cooling from the liquid state [15,16]. These MnSi striations always form perpendicular to the *c*-axis of the HMSs and these striations can cause serious damage at the HMS/MnSi interfaces during heating cycles. Furthermore, the existence of inhomogeneous boundaries would cause the electric conduction of the materials to deteriorate and should thus be avoided. Aoyama et al. [4] reported that a small amount of Ge substitution at the Si sites (∼0.5 at %) is effective in thinning and eventually dissipating the MnSi striations. Zhou et al. [5] also reported that Ge substitution was an effective approach to reduce the number of MnSi striations but that it was not possible to dissipate the striations completely, even at the vicinity of the Ge solubility limit.

Partial substitution of the Mn sites is also effective for dissipation of the MnSi striations. Miyazaki et al. [17] discovered that ∼2 at % substitution of V for Mn caused complete dissipation of the MnSi striations and considerable improvement in the electrical conductivity was also observed because of the synergistic effects of the disappearance of the scattering media and the increased numbers of hole carriers. They also observed that some of the X-ray diffraction (XRD) peaks of the materials split into doublets and shoulders around the composition at which the MnSi striations disappeared. These experimental facts correlate with each other and are crucial to further understanding of the physico-chemical and metallurgical aspects of HMS-based TE materials. To interrogate the crystal structure in detail, use of the superspace approach is necessary because HMSs have incommensurate composite structures that consist of two tetragonal subsystems for [Mn] and [Si], which have identical *a* and *b* axes but different *c* axes denoted by $c_{\mathrm{Mn}}$ and $c_{\mathrm{Si}}$. Using the *c*-axis length ratio γ = $c_{\mathrm{Mn}}$/$c_{\mathrm{Si}}$, the structural formula for HMSs can be represented by MnSi${}_{\gamma }$. In this study, we investigate the compositional changes in the crystal structure, dissipation of the MnSi striations, and the TE properties of the V- and Ge-substituted solid solutions.

## 2. Materials and Methods

Samples were prepared in an arc-melting furnace (GMAC-1100, GES ) under an Ar atmosphere using a tungsten-rod electrode and a water-cooled copper hearth. Appropriate amounts of Mn (99.99%), V (99.9%), Si (99.999%) and Ge (99.99%) reagents, based on the nominal compositions of (Mn${}_{1-x}$V${}_{x}$)Si${}_{1.74}$ or Mn(Si${}_{1-y}$Ge${}_{y}$)${}_{1.74}$, were melted four times and were turned over between runs to obtain full homogeneity. The button samples that were obtained were then crushed into small pieces and sealed in evacuated quartz tubes. Each tube was heated to 1473 K and this temperature was maintained for 8 h before being cooled down to 1373 K for another 100 h. The resulting samples were then furnace-cooled to room temperature. All ingots obtained consisted of highly-oriented nearly single crystals with relative densities of more than 93%. Each ingot was initially cut perpendicular to its growth direction into several pieces. The pieces that were cut from the center of each ingot were then cut further into the appropriate shapes required for the TE measurements, which are described below. XRD measurements were performed using Cu K${}_{\alpha }$ radiation (D8 Advance, Bruker AXS, Karlsruhe, Germany). Le Bail pattern fitting based on the (3+1)-dimensional superspace group *I*4${}_{1}$/amd(00γ)00ss [18] for the XRD patterns obtained was then performed using the Jana 2006 software [19].

The microstructures and elemental distributions were investigated using a scanning electron microscope (SEM; SU-8100, Hitachi, Tokyo, Japan) equipped for energy-dispersive spectroscopy (EDS). The sample orientation was evaluated based on electron backscatter diffraction (EBSD) patterns acquired using a field emission SEM (JSM-7100F, JEOL, Tokyo, Japan).

The Seebeck coefficient and the electrical conductivity were measured simultaneously in a He atmosphere over the range from room temperature to 1000 K using an automated thermoelectric tester (ZEM-3, ADVANCE RIKO, Kanagawa, Japan). The thermal conductivity was measured in a vacuum using a standard laser flash thermal constants analyzer (TC-7000H, ADVANCE RIKO, Kanagawa, Japan). The carrier concentration and mobility were evaluated using a Quantum Design physical property measurement system (PPMS, San Diego, CA, USA) operating at 293 K. All TE properties were measured along the same direction for all melt-grown ingots, but this does not mean that the measured direction is exactly parallel to a specific crystallographic direction in the samples, as will be described later in the paper.

## 3. Results and Discussion

### 3.1. XRD Patterns and Microstructures

**Figure 1:**
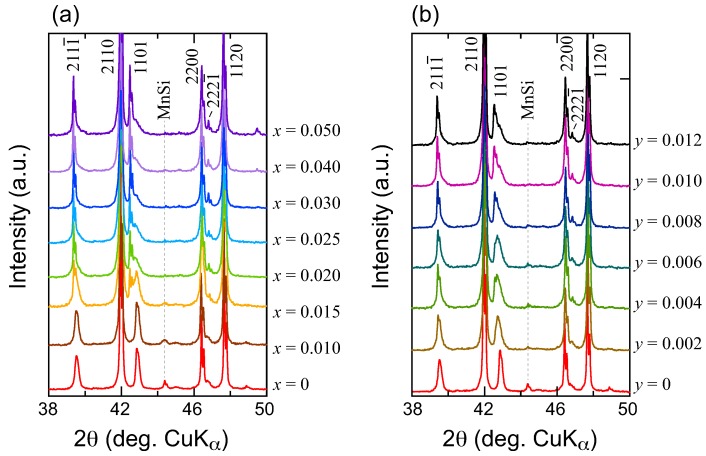
Enlarged powder X-ray diffraction (XRD) patterns of melt-grown samples with nominal compositions of (**a**) (Mn${}_{1-x}$V${}_{x}$)Si${}_{1.74}$ and (**b**) Mn(Si${}_{1-y}$Ge${}_{y}$)${}_{1.74}$.

Figure 1a,b represent XRD patterns with a 2θ range of 38–50${}^{\circ }$ for (Mn${}_{1-x}$V${}_{x}$)Si${}_{1.74}$ and Mn(Si${}_{1-y}$Ge${}_{y}$)${}_{1.74}$ samples, respectively; some of the data in (a) have already been published in the literature [17]. Four integers are required in order for the Bragg peaks to be indexed fully, the reflections with indices of hkl0 and hk0m originate from the [Mn] and [Si] subsystems, respectively, while those with the index of hklm are satellite reflections. The substitution-free (*x* = *y* = 0) samples comprise sharp and intense peaks with indices including 2110, 2200, and 1120, which originated from the [Mn] subsystem, broad but weaker peaks with indices such as 1101 that originated from the [Si] subsystem and satellite reflections with indices including 211$\bar{1}$. The main MnSi reflections are also recognized at 2θ∼44.4${}^{\circ }$. As the V-content *x* increases, the peaks belonging to the [Mn] subsystem do not shift appreciably, but the 1101 and satellite peaks change their shapes dramatically. At *x* = 0.015, where the main MnSi peak almost fades into the background, the 1101 peak splits into a lower-angled but sharper doublet of $K_{\alpha 1}$ and $K_{\alpha 2}$, plus a higher-angled and broader peak. Transmission electron microscopy (TEM) observations indicate that the nanostructure of this sample consists of two types of domains: domains composed of tens of nanometer-scale highly strained arrangements of Si atoms (*strained domains*) that are randomly embedded in matrices composed of regularly arranged Si atoms (*regular domains*), while the arrangement of the Mn atoms remains highly ordered throughout the sample. The regular domains yield the lower-angled and sharper peaks, while the strained domains cause the higher-angled and broader peaks (shoulders) that appear in the XRD patterns around 2θ = 43${}^{\circ }$. The relative numbers of these domains can be evaluated by integrating the intensities of the corresponding peaks. Therefore, the shape of the 1101 peak splitting can serve as a measure of the related nanostructures of the samples. More details of the TEM observations, including the evolution of several elemental substitutions, will be published elsewhere. Hereafter, we refer to this peak splitting of the 1101 peak as *domain separation*. As *x* increases, the relative intensity of the regular domains becomes dominant. The disappearance of the main MnSi peak appears to coincide with the occurrence of the domain separation. No discernible changes in the peak position and shape were recognized for x≥ 0.030.

In contrast to the V-substituted series, domain separation is not significant in the Mn(Si${}_{1-y}$Ge${}_{y}$)${}_{1.74}$ solid solution within the Ge solubility limit at the Si sites. The broader 1101 peaks (when compared with those of the V-substituted series) imply that the nanostructure comprises more uniformly distributed domains that are composed of mildly strained arrangements of partly Ge-substituted Si atoms. It also appears that slight traces of the main MnSi peaks remain even at *y* = 0.012.

**Figure 2:**
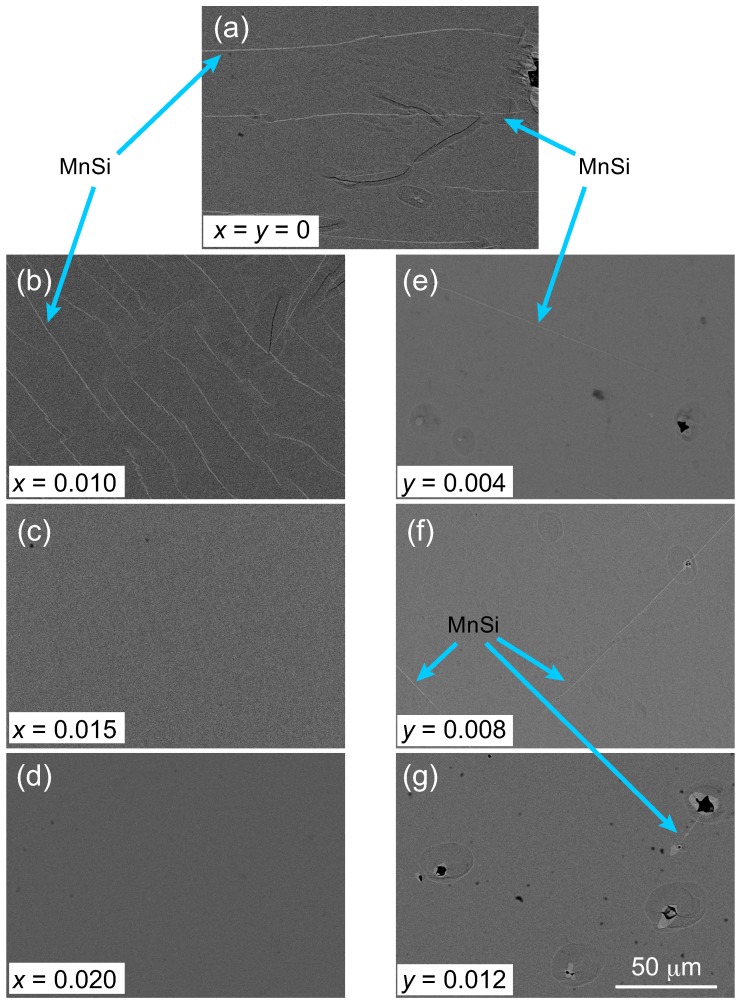
Scanning electron microscope (SEM) images of (**a**) substitution-free MnSi${}_{1.74}$, (**b**–**d**) V-substituted (Mn${}_{1-x}$V${}_{x}$)Si${}_{1.74}$, and (**e**–**g**) Ge-substituted Mn(Si${}_{1-y}$Ge${}_{y}$)${}_{1.74}$ samples.

Figure 2a–g shows SEM images of the substitution-free, V-substituted and Ge-substituted samples, respectively. The bright lines that run roughly parallel to each other that can be observed in (a) are the MnSi striations. The number of these MnSi striations increases with increasing *x* , although their thicknesses appear to decrease, as shown in (b). The MnSi striations disappear completely with further increases in *x*, as shown in (c) and (d), which coincides with the disappearance of the main XRD peak for MnSi that is shown in Figure 1. In the case of the Ge-solid solution, a small amount of Ge substitution effectively thins and reduces the number of MnSi striations, as shown in (e). However, the MnSi striations cannot be removed completely, even in the cases shown in (f) and (g); this is similar to the results reported by Zhou et al. in the literature [5].

### 3.2. Lattice Parameters

**Figure 3:**
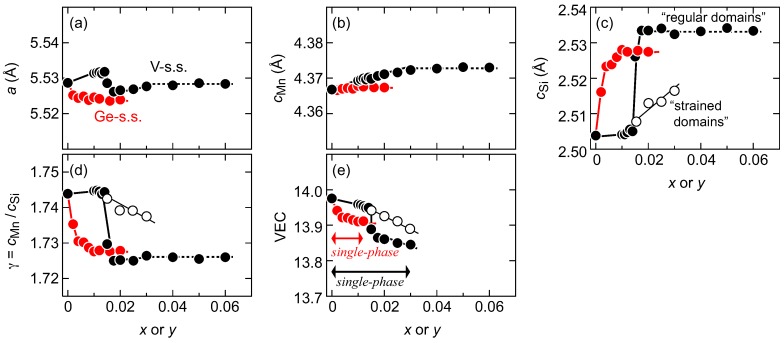
Effects of V-content (*x*) or Ge-content (*y*) on the lattice parameters of (**a**) *a*, (**b**) $c_{\mathrm{Mn}}$, (**c**) $c_{\mathrm{Si}}$ and (**d**) γ for either the (Mn${}_{1-x}$V${}_{x}$)Si${}_{1.74}$ solid solution (V-s.s.) or Mn(Si${}_{1-y}$Ge${}_{y}$)${}_{1.74}$ solid solution (Ge-s.s.). The estimated valence electron counts per number of transition metals (VEC) in the single-phase samples are plotted versus *x* or *y*, as shown in (**e**). Black and red closed circles in each panel represent the refined data using peaks belonging to regular domains of V-s.s. and Ge-s.s., respectively, while open circles are those estimated from strained domains in the V-s.s.

Figure 3a–d illustrates the effects of the nominal V-content *x* or the Ge-content *y* on the lattice parameters of *a*, $c_{\mathrm{Mn}}$, $c_{\mathrm{Si}}$ and γ, as refined using the Le Bail fitting, for either the (Mn${}_{1-x}$V${}_{x}$)Si${}_{1.74}$ or Mn(Si${}_{1-y}$Ge${}_{y}$)${}_{1.74}$ samples. We confirmed that the averaged chemical composition of each sample well agreed with that of the nominal one. Hence, we hereafter use the nominal compositions to describe samples. The standard deviation for each parameter is within the symbol size depicted for all data. Because the atomic radius of V ($r_{V}$ = 1.321 Å) [20] is greater than that of Mn ($r_{\mathrm{Mn}}$ = 1.24 Å), and the atomic radius of Ge ($r_{\mathrm{Ge}}$ = 1.225 Å) is also larger than that of Si ($r_{\mathrm{Si}}$ = 1.17 Å), partial substitution with V or Ge is expected to expand the [Mn] or [Si] subsystems, respectively. However, with the exception of the $c_{\mathrm{Mn}}$-axis length, the lattice parameters exhibit unusual behavior with respect to *x* and *y*, in that the samples do not obey the well-known Vegard’s rule. With a small amount of V-substitution up to *x* = 0.014, the *a*-axis length increases with increasing *x*, but then suddenly decreases for values of *x* of up to 0.018 before turning again to increase up to a value of *x* = 0.030. However, further increases in *x* do not cause the *a*-axis length to change appreciably. In contrast, the $c_{\mathrm{Mn}}$-axis length increases almost linearly with increasing *x* up to *x* = 0.030. Another unusual change is also observed in the $c_{\mathrm{Si}}$-axis length; this length remains at an almost constant value of ∼2.505 Å up to *x* = 0.014, but is then suddenly elongated to ∼2.535 Å at *x* ≥ 0.018. These sudden changes occur at the material composition where the domain separation that was observed in Figure 1 takes place. If the $c_{\mathrm{Si}}$-axis length estimated from the higher-angled and broader peaks is plotted, it can be represented as shown by the open circles in (c). The coexistence of the longer (regular domains) and shorter (strained domains) $c_{\mathrm{Si}}$-axis lengths will affect both carrier and phonon transport; this point will be highlighted in Section 3.4. By fitting only the lower-angled and sharper peaks, we obtain the γ values indicated by the filled black circles shown in (d). Typical melt grown samples with *x* = *y* = 0 have a γ value of slightly more than 1.74. The 1101 peak splitting reduces the γ value of the majority (regular) domains to ∼1.725, where these domains coexist with the minority (strained) domains of ∼1.74. Because all the lattice parameters remain unchanged at *x* ≥ 0.030, the solubility limit for V should be 0.030.

The Ge-solid solution shows different behavior to that demonstrated by the V-series. A tiny amount of Ge can effectively shrink the *a*-axis but causes slight expansion of the $c_{\mathrm{Mn}}$-axis, as shown in (a) and (b). When compared with the small change in size in the [Mn] subsystem, the $c_{\mathrm{Si}}$-axis expands more markedly here with small amounts of Ge-substitution ranging up to *y* = 0.012, as shown in (c). However, no remarkable increases are observed with any further increase in *y*. From (a)–(c), the Ge solubility limit can be estimated to be *y* = 0.012, which is comparable to previously reported values [5]. As per the V-substitution case, the Ge-substitution also causes a sudden reduction in γ; ∼0.5 at % of Ge-substitution reduces the γ value significantly to ∼1.73, while the samples with *x* = 0.012 yield γ values of less than 1.73.

In Figure 3e, the VEC value is plotted against *x* or *y* for single-phase samples of either (Mn${}_{1-x}$V${}_{x}$)Si${}_{1.74}$ or Mn(Si${}_{1-y}$Ge${}_{y}$)${}_{1.74}$ solid solutions. The VEC value is known to represent the relative shift in the Fermi energy ($E_{F}$) in the electronic structure; VEC is an abbreviation for the valence electron counts per number of transition metals [21]. When the VEC value is less than 14, $E_{F}$ is shifted in the valence band and the sample will then exhibit p-type behavior; smaller VEC values correspond to higher hole concentrations. For the HMS-based solid solutions presented here, the VEC can be expressed as follows: 7(1 −*x*) + 5*x* + 4{(1 −*y*) + *y*)}γ. In this expression, the numbers 7, 5 and 4 represent the valence electron numbers of Mn, V and Si(Ge), respectively. Because the valence electron numbers of Si and Ge are equal, the above formula can be rewritten as 7(1 −*x*) + 5*x* + 4γ = 7 − 2*x* + 4γ, which is independent of *y*. The substitution-free sample has a VEC value of ∼13.97, i.e., slightly less than 14, which is a typical value for HMSs. However, with increasing *x*, the VEC suddenly drops to ∼13.9 at approximately *x* = 0.015 and reaches ∼13.85 at the V solubility limit with *x* = 0.030. Ge substitution is quite effective in reducing the VEC; only a small amount of *y* introduces multiple hole carriers but further Ge substitution cannot drive the VEC below 13.91. These results indicate that samples with values of *x* = 0.015–0.030 will be more electrically conductive and thus exhibit higher σ values than the Ge-substituted samples.

### 3.3. Thermoelectric Properties

**Figure 4:**
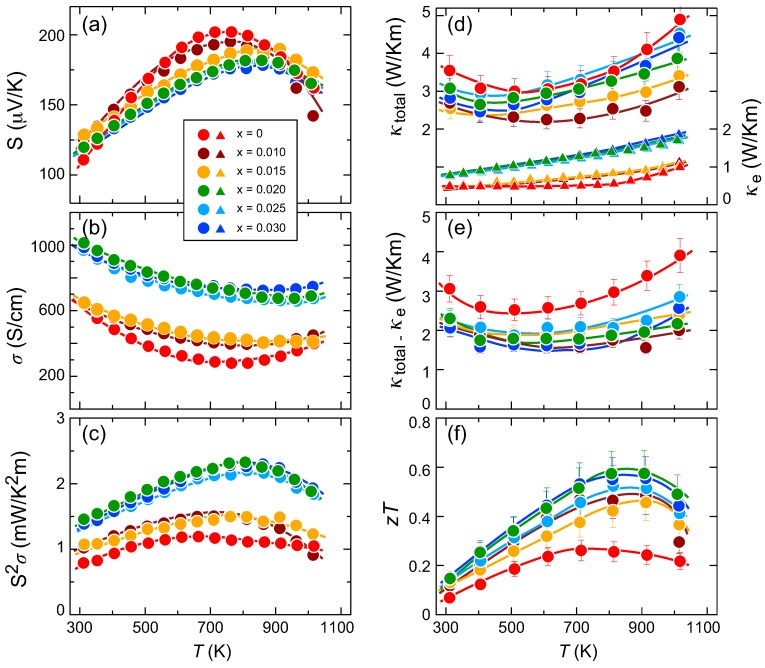
Temperature dependences of (**a**) Seebeck coefficient *S*, (**b**) electrical conductivity σ, (**c**) power factor $S^{2}\sigma$, (**d**) total thermal conductivity $\kappa_{\mathrm{total}}$ and its carrier contribution $\kappa_{e}$, (**e**) $\kappa_{\mathrm{total}}$-$\kappa_{e}$ and (**f**) the dimensionless figure of merit of partially V-substituted samples with a nominal composition of (Mn${}_{1-x}$V${}_{x}$)Si${}_{1.74}$.

Figure 4a–c illustrate the temperature dependences of (a) the Seebeck coefficient *S*, (b) the electrical conductivity σ and (c) the TE power factor $S^{2}\sigma$ for partially V-substituted samples composed of (Mn${}_{1-x}$V${}_{x}$)Si${}_{1.74}$. Note here the sample-cutting directions and the actual crystallographic directions of the samples. As described in the *Materials and Methods* section, all TE properties were measured along the same direction for the melt-grown ingots. The EBSD analyses show that the measured direction deviates by 0–20${}^{\circ }$, depending on the individual sample, from the crystallographic *a*-*b* plane. The results of our preliminary experiments on the effects of crystallographic orientation on the TE properties indicate that an angular deviation on this scale is negligibly small, in spite of the highly anisotropic crystal structure, and all the presented samples can thus be treated without any correction for anisotropy. Moreover, as the amount of secondary phase MnSi is estimated to be less than 2 at %, the effect of such a secondary phase on TE properties should be also negligible.

The substitution-free sample exhibits the maximum *S* value of $S_{\max}$∼200 μV/K at $T_{\max}$∼700 K, which is a typical value for a single-crystal sample when measured parallel to the *a*-*b* plane. With increasing *x*, $S_{\max}$ decreases moderately while $T_{\max}$ is gradually shifted toward a higher *T* because of the increase in the hole concentration. A large reduction in *S* at around *T* = 700 K, in the range between *x* = 0 and 0.015, corresponds to the sudden reduction in the VEC that is associated with domain separation. As *x* continues to increase from 0.020 to 0.030, the *S* values nearly overlap because the reduction in the VEC is less marked against *x*. The σ value shown in (b) gradually increases up to *x* = 0.015 before showing a sudden increase for samples with *x* = 0.020–0.030, which is also related to the domain separation. Because of the results shown in (a) and (b), the TE power factor is almost doubled because $S^{2}\sigma$ = 2.3–2.4 mW/K${}^{2}$m at 800 K for the samples with *x* = 0.020–0.030 when compared with the substitution-free sample, for which $S^{2}\sigma$ = 1.0–1.1 mW/K${}^{2}$m at 600 K.

Figure 4d–f plot the temperature dependences of (d) the total thermal conductivity $\kappa_{\mathrm{total}}$ and its carrier contribution $\kappa_{e}$, (e) $\kappa_{\mathrm{total}}$-$\kappa_{e}$ and (f) the dimensionless figure of merit of the partially V-substituted samples of (Mn${}_{1-x}$V${}_{x}$)Si${}_{1.74}$. The $\kappa_{e}$ terms were simply calculated using the Wiedemann-Franz law of $\kappa_{e}$ = *L*σ*T*, where *L* = 2.45 × 10${}^{-8}$ WΩK${}^{-2}$ is the Lorenz number. The total thermal conductivity appears to show little *x*-dependence, while the $\kappa_{e}$ value reflects the electrical conductivity shown in (b). The lattice thermal conductivity $\kappa_{L}$ can be evaluated by subtracting $\kappa_{e}$ from $\kappa_{\mathrm{total}}$, as plotted in (e). At lower values of *T*, where the $\kappa_{\mathrm{total}}$-$\kappa_{e}$ product simply decreases versus *T*, this may represent the temperature dependence of $\kappa_{L}$. However, all the samples show upturns at a specific *T*, which may be related to the bipolar diffusion effect. We therefore refrain from assigning the behavior of $\kappa_{\mathrm{total}}$-$\kappa_{e}$ to represent that of $\kappa_{L}$. What we can say is that little effect from the domain separation exists here to effectively reduce $\kappa_{L}$ because the typical size of the domains involved is several tens of nm. If we can control the sizes and distributions of the regular and strained domains appropriately, we should be able to observe a certain reduction in $\kappa_{L}$ in the HMS-based solid solutions presented here because the effective phonon mean free path (MFP) is estimated to be ∼10 nm [22]. As a result of (c) and (d), we can obtain satisfactory TE performance from the samples with *x* = 0.020-0.030, where zT> 0.5 at 800–900 K, as compared with the *x* = 0 sample, where zT = 0.27.

**Figure 5:**
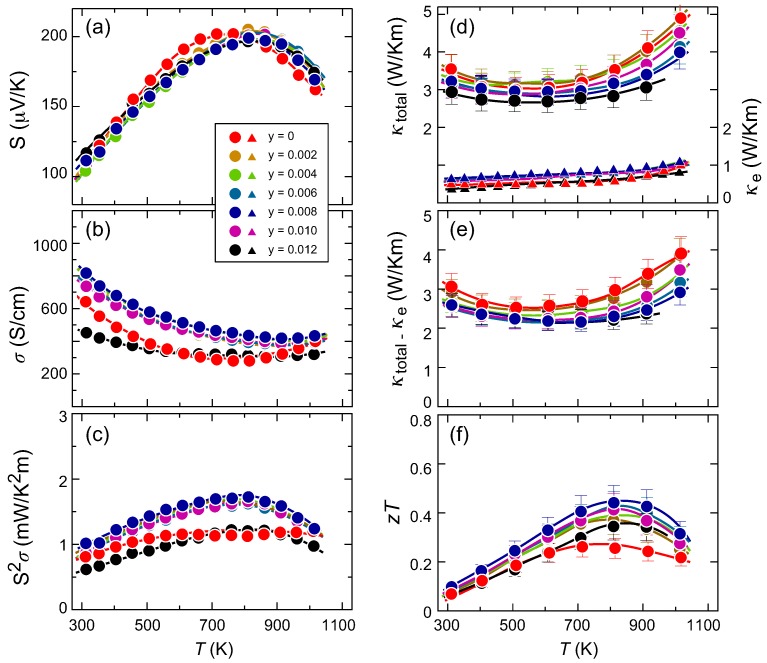
Temperature dependences of (**a**) the Seebeck coefficient *S*, (**b**) electrical conductivity σ, (**c**) power factor $S^{2}\sigma$, (**d**) total thermal conductivity $\kappa_{\mathrm{total}}$ and its carrier contribution $\kappa_{e}$, (**e**) $\kappa_{\mathrm{total}}$-$\kappa_{e}$ and (**f**) the dimensionless figure of merit of the partially Ge-substituted samples with a nominal composition of Mn(Si${}_{1-y}$Ge${}_{y}$)${}_{1.74}$.

Figure 5a–c represent the temperature dependences of (a) the Seebeck coefficient *S*, (b) the electrical conductivity σ and (c) the TE power factor $S^{2}\sigma$ of the partially Ge-substituted samples of Mn(Si${}_{1-y}$Ge${}_{y}$)${}_{1.74}$. Unlike the previous V-substituted solid solutions, the Ge series show little *y*-dependence on *S*, with the exception of a small shift in $T_{\max}$ from 700 K at *y* = 0 to 800 K for the *y* = 0.002–0.012 samples. This small change is likely to be related to the reduction of the VEC from 13.97 for *y* = 0 to 13.91 for *y* = 0.004–0.012. Reflecting this small change in the VEC, the σ value shows only a small enhancement from the Ge-free samples to the samples with *y* = 0.002–0.010. The reason why the sample with *y* = 0.012 shows poor electrical conductivity is unclear, but it is possible that the sample may contain microcracks that cause σ to deteriorate. Based on (a) and (b), the power factor shows the maximum value of $S^{2}\sigma$ = 1.7 mW/K${}^{2}$m at 800 K for samples with *y* = 0.008.

Figure 5d–f plot the temperature dependences of (d) the total thermal conductivity $\kappa_{\mathrm{total}}$ and its carrier contribution $\kappa_{e}$, (e) $\kappa_{\mathrm{total}}$-$\kappa_{e}$ and (f) the dimensionless figure of merit of the partially Ge-substituted samples of Mn(Si${}_{1-y}$Ge${}_{y}$)${}_{1.74}$. In a manner similar to that of the V-substituted samples, no remarkable differences are recognized in any of the samples based on consideration of the estimated errors for (d) and (e). The zT value reaches its highest for the sample with *y* = 0.008, with a value that slightly exceeds 0.44 at 800 K. While this increase is only a relatively small amount, where zT = 0.27 (*y* = 0) to 0.44, it would be surprising to note that the ∼1.2 at % substitution of Ge effectively improves the microstructure and thus enhances the electrical conduction. The effects of increasing *y* on *S* on σ and thus on $S^{2}\sigma$ are comparable to those reported in the literature [4,5,6], but one apparent difference occurs in the κ values, which are roughly ∼2.0–2.5 W/Km in the polycrystalline samples and yield a higher zT of 0.4–0.7 at 800 K, as compared with the even higher κ values of the near-single-crystal samples presented here, which lead to lower zT values.

### 3.4. Effects of Domain Separation on TE Properties

**Figure 6:**
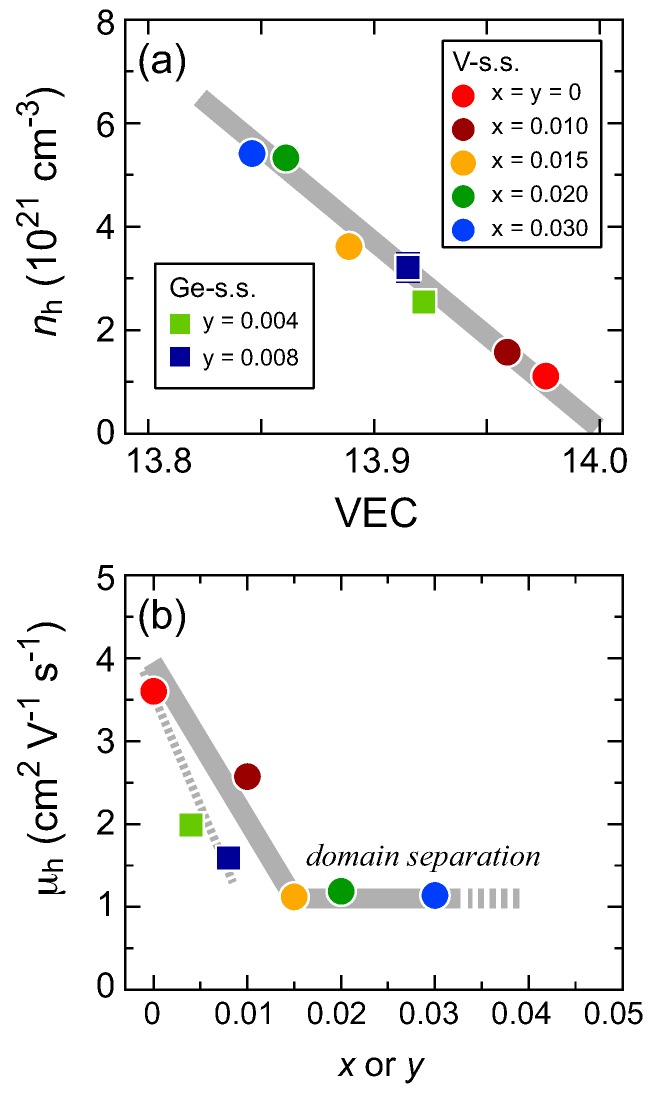
(**a**) Hole carrier concentration $n_{h}$ at 293 K plotted versus the VEC of the presented (Mn${}_{1-x}$V${}_{x}$)Si${}_{1.74}$ solid solution (V-s.s.) and Mn(Si${}_{1-y}$Ge${}_{y}$)${}_{1.74}$ solid solution (Ge-s.s.). (**b**) Hole mobility $\mu_{h}$ at 293 K plotted versus *x* or *y* for the same samples.

To investigate the effects of the domain separation on the TE properties, Hall measurements were performed. In Figure 6a, we plot the hole carrier concentration $n_{h}$ at 293 K versus the VEC of the regular domains. The figure contains data for both the V- and Ge-substituted solid solutions. The thick gray line represents the relationship between the VEC and the calculated $n_{h}$, which represents the product of $n_{h}$ = 4(14 − VEC)/$V_{\mathrm{Mn}}$, where $V_{\mathrm{Mn}}$ represents the unit volume of the [Mn] subsystem. Because all the data sit excellently along the gray line, $n_{h}$ at 293 K can be determined accurately using the VEC values of the HMS-based solid solutions. Without any substitutions, the sample has $n_{h}$ ∼1 × 10${}^{21}$ cm${}^{-3}$ at 293 K, which is consistent with previously reported values [21,23]. As observed in Figure 3e, any increase in *x* or *y*, even when the amount is less than 2 at %, leads to a significant reduction in the VEC, i.e., it effectively represents doping with hole carriers. The $n_{h}$ value is increased by three times with a < 1 at % substitution of Ge for Si, while the 2 at % substitution of V for Mn yields a five-fold increase in $n_{h}$ at 293 K.

Figure 6b represents the hole mobility $\mu_{h}$ at 293 K plotted versus the *x* or *y* values of V- or Ge-substituted solid solutions. Partial substitution apparently reduces $\mu_{h}$, but the domain separation process appears to prevent any further deterioration of the carrier mobility in the V-series; the samples with 0.015 ≤ *x* ≤ 0.030 have a near-constant $\mu_{h}$ of ∼1.0 cm${}^{2}$V${}^{-1}$s${}^{-1}$. This is not the case for the Ge-solid solution because $\mu_{h}$ simply decreases with increasing *y*. The Ge solubility limit (*y* = 0.012) may be insufficient to yield the distinct 1101 peak splitting in the XRD patterns that corresponds to the domain separation process. The domain-separated samples of the V-series show a higher σ because of the higher $n_{h}$ and near-constant $\mu_{h}$ that occur around room temperature. Because $n_{h}$ is known to remain almost constant versus *T* until the bipolar diffusion effect occurs in the HMS-based samples [23], a gradual decrease in σ versus temperature up to the *T*∼800–900 K limit is mainly caused by the decrease in $\mu_{h}$.

Finally, one critical problem remains: the question of why the domain separation occurs in the V-substituted samples. Domain separation has been found to occur at a specific composition at which the MnSi striations disappear and thus these phenomena must be strongly correlated. To address this problem, we first consider the formation mechanism of the MnSi striations. As previously known [15,16,24], the MnSi striations are formed as primary crystals during crystallization from a liquid state. They always form perpendicular to the *c*-axis of the HMS. This leads to the question of how the primary MnSi crystals know the *c*-axis direction of the HMS, which will nucleate and grow *after the MnSi formation*. We believe that the formation of the MnSi striations mainly originates from the difference in the coefficient of thermal expansion (CTE) values of the [Mn] and [Si] subsystems that are oriented parallel to the *c*-axis during heat cycling. The CTE of the [Si] subsystem becomes three times larger than that of the [Mn] subsystem at higher temperatures of *T* > 800 K, but the CTEs are equal around room temperature [25]. This change in the CTE causes a subsequent change in γ, from ∼1.74 at lower temperatures to ∼1.72 at higher temperatures, and some of the Si atoms must move out of the structure towards a location with greater energy stability along the *c*-axis, upon cooling. The MnSi striations may then arise from such a mechanism. If we can control the γ value to ensure that it is *T*-independent, we should observe no MnSi striations. The V-substituted solid solution could be a suitable case and its γ value is actually close to 1.72 even at room temperature. In the actual samples, however, it appears to be impossible to form domains that comprise only γ ∼1.72 for certain energy-related reasons and it may be necessary for some parts to exist as a highly disordered arrangement of Si atoms. The existing ratio of regular to strained domains can be roughly estimated to be 80:20 based on the relative intensities of the 1101 peaks from XRD. This could be the reason why the carrier mobility is not disturbed by domain separation around room temperature. To confirm our proposed scenario, further studies using TEM and detailed crystal structure analysis while varying the temperature may be necessary.

## 4. Conclusions

We have performed partial substitutions of V for the Mn sites or Ge for the Si sites of HMSs to investigate their crystal structures and TE properties. Both substitutions are confirmed to be effective approaches to suppress the formation of the MnSi striations. However, the dissipation effect is much stronger in the case of the V-substitutions, which eliminate the MnSi striations completely. We have also proposed a possible scenario to help us to understand the formation (dissipation) of the MnSi striations, which will lead to the production of superb HMS-based TE materials.
